# Female predominance in chronic cough: biological sex–related factors, mechanisms, and therapeutic targets—a narrative review

**DOI:** 10.3389/fmed.2026.1790629

**Published:** 2026-03-12

**Authors:** Aibo Zheng, Kai Sun, Wenjun Li, Zhiyu Chen, Yuting Li, Wei Wu, Jie Gan

**Affiliations:** Department of Respiratory and Critical Care Medicine, Zigong Fourth People’s Hospital, Zigong, Sichuan, China

**Keywords:** biological sex differences, chronic cough, cough hypersensitivity, P2X3 receptor antagonists, TRP channels

## Abstract

Chronic cough—defined in adults as cough lasting ≥8 weeks—shows higher prevalence in women. Accumulating evidence supports a cough hypersensitivity phenotype with peripheral and central drivers that may vary by biological sex. Evidence-based evaluation emphasizes identification and treatment of common etiologies (upper-airway cough syndrome, cough-variant asthma or eosinophilic bronchitis, and reflux-related cough) and recognition of refractory chronic cough (RCC) and unexplained chronic cough (UCC) when symptoms persist despite optimized care. Therapeutic options include behavioral and neuromodulatory approaches as well as selective antagonists of receptors, purinergic P2X (P2X3), which can reduce objective cough frequency, although taste disturbance may occur. This review summarizes factors that may contribute to female predominance—including hormonal and immune modulators, neuro-sensory processing, comorbidities, and environmental triggers—and appraises the therapeutic landscape with attention to benefits, harms, and sex-aware evidence gaps. We outline priorities for research, including standardized outcomes, sex-stratified analyses, and patient-centered measures to improve diagnosis and management for women with chronic cough. Abbreviations: ERS, European Respiratory Society; BTS, British Thoracic Society; RCC, refractory chronic cough; UCC, unexplained chronic cough. Aims: This article reviews the factors associated with the female prevalence of chronic cough, potential mechanisms, and targets for therapy. In this review, the term “sex” refers to biological sex differences, whereas sociocultural gender-related factors are discussed only when relevant.

## Introduction

In adults, chronic cough is conventionally defined as cough lasting ≥8 weeks, as recommended by the European Respiratory Society (ERS) 2020 guidelines, with similar thresholds adopted in CHEST and British Thoracic Society (BTS) statements (1–3). Evaluation should be cause-directed and evidence-based, typically including a focused history (e.g., ACE-inhibitors), chest radiograph, spirometry/bronchodilator testing, and directed work-up for common etiologies, with referral thresholds for refractory/unexplained chronic cough. After excluding abnormal chest radiography and medication-induced cough, common causes include CVA, eosinophilic bronchitis, UACS and GERD (4). Women comprise roughly two-thirds of chronic cough populations in many series, and middle-aged or older women are over-represented among refractory or unexplained cases (5). Potential explanations include cyclical biological sex-hormone effects on airway sensory pathways, distinct immune responses, and differential exposure or susceptibility to environmental triggers. These features have implications for diagnosis, therapy and trial design (6, 7).

Importantly, female predominance is especially pronounced in peri- and postmenopausal age groups in many referral cohorts. Accordingly, throughout this review we distinguish hormonally dynamic states (e.g., menstrual cycle and pregnancy) from the menopausal transition, and we emphasize mechanisms that remain biologically plausible when ovarian hormone levels are chronically low (e.g., altered airway–vagal sensory excitability, central cough network modulation, comorbidity clustering, and immunometabolic changes).

Compared with the prior review by Bai et al. (8) that summarized early evidence for female- predominant cough sensitivity, our review (i) integrates post-2021 guidance and clinical statements, (ii) incorporates central-neurobiology and neuroimaging findings relevant to cough control, and (iii) critically appraises the expanding evidence on selective P2X3 antagonists (e.g., gefapixant phase 3; eliapixant phase 2b; camlipixant phase 2b) with attention to safety signals such as taste disturbance and implications for women.

Aims of this Review. This narrative review aims to (i) synthesize contemporary epidemiology of chronic cough with attention to female predominance; (ii) summarize biological sex-related mechanisms across peripheral, central, immunologic, and environmental pathways; (iii) appraise current and emerging therapies (including P2X3 antagonists) with relevance to women; and (iv) highlight gaps, female-focused research priorities, and implications for evidence-based practice.

## Epidemiology of chronic and female predominance

This section summarizes the global epidemiology of chronic cough with particular emphasis on the consistent female predominance observed in community-based cohorts. We review prevalence patterns across regions, age groups, and healthcare settings, and discuss methodological considerations that may influence sex distribution estimates. These data provide the clinical foundation for exploring biological, neurophysiological, and sociocultural mechanisms underlying female-predominant cough.

The global prevalence of chronic cough in adults has been estimated at 9.6%, with women accounting for approximately 66–73% of affected individuals (5). Although internet-based surveys generally yield lower response rates than traditional face-to-face investigations, response rates in the range of 25–30% are commonly reported and are considered acceptable for population-based epidemiological research (9).

Data from the Tasmanian Longitudinal Health Study (TAHS) further highlight both the heterogeneity and sex distribution of chronic cough at the population level. In TAHS, nearly half of chronic cough cases (46.4%) were reported to have no clearly identifiable etiology (10), underscoring the contribution of unexplained or hypersensitivity-related phenotypes in the community. Complementing this observation, a recent TAHS phenotype analysis (38109918) using latent subclass modeling identified distinct cough phenotypes, including an “allergic cough” subclass characterized by atopic features and heightened airway sensitivity, which demonstrated female predominance. These findings suggest that female overrepresentation in chronic cough may be enriched within specific biologically defined subgroups rather than uniformly distributed across all presentations. Similar sex patterns have been observed in other population-based datasets; for example, the Italian National Health and Wellness Survey (NHWS) reported a lifetime prevalence of 9.2% and a 12-month prevalence of 6.3%, with chronic cough occurring more frequently in women, individuals with a history of smoking, and residents of southern regions of Italy (11).

In the United States, the prevalence of chronic cough persisting for more than 8 weeks is estimated at around 10%, with women comprising 52.8% of affected patients (12). Similarly, a large prospective cohort study from Rotterdam reported a higher prevalence of chronic cough among women aged 70 years and older compared with men (3.4% vs. 2.0%), corresponding to a prevalence ratio of 1.73 (95% CI 1.12–2.66) (13).

Evidence from China’s national health insurance database further supports a female predominance, with women accounting for 55.24% of chronic cough cases between 2015 and 2017. However, regional variations have been observed. In cities such as Guangzhou and Lanzhou, the biological sex distribution appears more balanced, which may be attributable to differences in age structure, smoking prevalence, and environmental exposures. In particular, pollen and dust mite sensitisation in Guangzhou, as well as industrial pollution and sandstorms in Lanzhou, may contribute to the observed heterogeneity in disease distribution (1, 14).

## Guideline-based diagnostic approach and phenotyping

This section outlines current diagnostic frameworks for chronic cough and examines whether sex-related differences influence clinical presentation, comorbidity patterns, or diagnostic pathways. We distinguish between treatable traits and cough hypersensitivity phenotypes, highlighting how symptom perception, laryngeal sensitivity, and health-seeking behavior may vary between women and men. Clarifying phenotypic heterogeneity is essential for understanding apparent sex disparities and optimizing personalized evaluation strategies.

### Chronic cough after COVID-19 infection

It is estimated that 20–30% of patients develop a chronic cough following a SARS-CoV-2 infection, with 2.5% of patients still experiencing cough symptoms 1 year after the initial infection (15). A single-center study in Japan found that the prevalence of chronic cough following a SARS-CoV-2 infection increased from 13 to 24% (16). Another study reported that 47.1% of these patients were newly diagnosed with asthma (17). These findings suggest that the SARS-CoV-2 virus causes damage to the respiratory system and may lead to long-term respiratory issues, potentially resulting in new disease diagnoses. Notably, the incidence of chronic cough after a diagnosis of SARS-CoV-2 is significantly higher in female patients than in males, which may be related to the heightened sensitivity of the female immune system and hormonal fluctuations (18). Further investigation into this phenomenon could help to optimize treatment strategies for female patients with chronic cough and improve their quality of life.

### Risk factors

Environmental exposures are increasingly recognized as important contributors to chronic cough in certain populations. Air pollution, including combustion-derived particles and polycyclic aromatic hydrocarbons (PAHs), has been associated with increased respiratory symptoms in epidemiological studies, including chronic cough in some cohorts. Indoor air quality, particularly elevated PM2.5 concentrations, may further exacerbate airway irritation, especially in settings with prolonged exposure. A recent systematic review of risk factors for chronic cough (34658107) further consolidates evidence linking environmental pollutants, smoking exposure, and underlying airway disease to increased chronic cough prevalence across diverse populations (19). However, the magnitude and consistency of these associations vary across studies and populations.

Occupational inhalational exposures, such as metalworking fluids and isocyanates, are established triggers of airway inflammation and have been linked to non-allergic eosinophilic bronchitis (20), a condition commonly presenting with chronic cough. Prolonged exposure to these agents may promote airway epithelial injury and inflammatory responses relevant to cough persistence.

Heavy metal exposure has also been investigated in respiratory health. Experimental and cross-sectional studies suggest that cadmium exposure can increase oxidative stress and promote inflammatory signaling in bronchial epithelial cells, including enhanced reactive oxygen species (ROS) production and activation of apoptosis-related pathways (21). While these mechanisms are biologically relevant to airway injury, direct causal relationships between cadmium exposure and chronic cough require further clarification.

Smoking remains one of the most consistently reported risk factors for chronic cough. Population-based studies demonstrate higher prevalence of chronic cough among smokers compared with non-smokers (22, 23). Chronic respiratory diseases, including chronic obstructive pulmonary disease (COPD) and asthma, are also strongly associated with persistent cough symptoms (24, 25). Environmental irritants such as tobacco smoke, dust, and noxious gases may further amplify cough susceptibility (26).

Comorbid conditions, including chronic lung disease, metabolic disorders, and mood disorders, have been associated with increased chronic cough risk in observational analyses (15). Similarly, demographic characteristics such as age and ethnicity have been reported to influence cough prevalence or recurrence in some cohorts (15), although these associations may be context-dependent.

Emerging metabolic markers have also been explored. The non–high-density lipoprotein cholesterol-to–high-density lipoprotein cholesterol ratio (NHHR) has been investigated as a potential indicator associated with chronic cough risk in preliminary studies (27); however, this finding remains exploratory and requires validation in independent populations.

Importantly, environmental exposures may interact with biological sex–related mechanisms. Combustion-derived particles and oxidant gases can induce airway epithelial injury, oxidative stress, and neurogenic inflammation, including activation of TRP channels implicated in cough reflex sensitisation. Experimental and epidemiological data suggest that sex hormones may modulate inflammatory and sensory neural responses to inhaled irritants. Thus, observed female predominance in chronic cough in certain polluted or high-smoking environments may reflect not only differences in exposure burden but also potential biological responsiveness. Nonetheless, direct mechanistic studies examining sex-specific pollutant–cough interactions remain limited.

**Figure 1:**
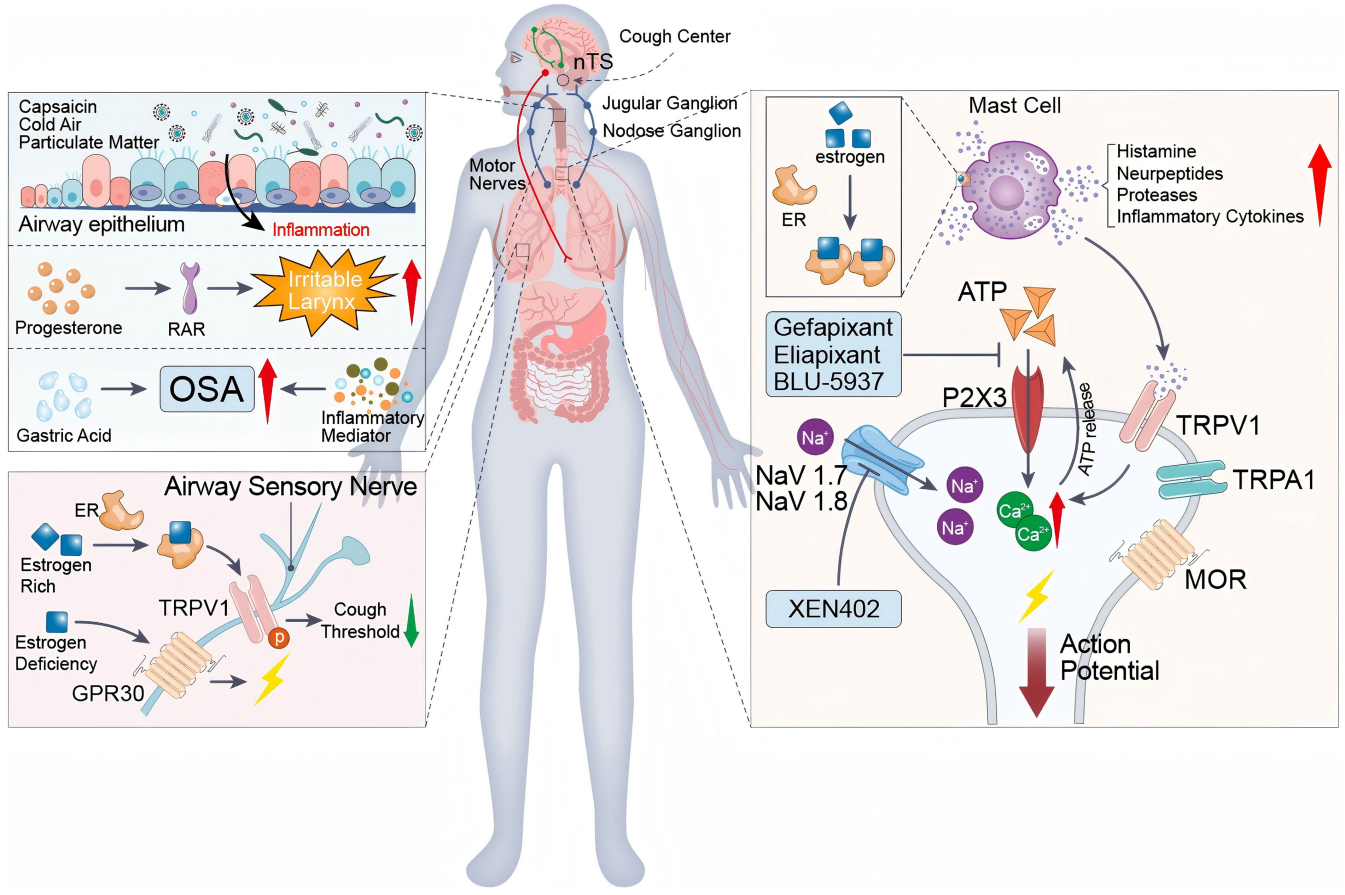
Mechanistic landscape of chronic cough in women. Schematic of female-predominant drivers of cough hypersensitivity and therapeutic targets: airway epithelial inflammation and irritant stimuli; estrogen/GPER modulation of TRPV1; brainstem pathways (nucleus tractus solitarius); peripheral terminals with ATP–P2X_3_ and TRPV1/TRPA1 signaling and NaV1.7/NaV1.8-dependent firing; and inhibitory *μ*-opioid receptor tone. Candidate antitussives act at defined nodes (for example, P2X_3_ antagonists and NaV blockade). The figure was created *de novo* by the authors using Adobe Illustrator, and no previously published material was reused.

Female predominance in chronic cough—most evident in middle-aged cohorts—has consistent epidemiologic support. Mechanistically, women demonstrate higher cough reflex sensitivity and heightened activation of peripheral sensory channels (for example TRPV1 and P2X3) on airway–vagal afferents, and experimental studies show TRPV1–P2X3 co-expression and functional crosstalk that can shape primary sensory-neuron excitability (28–31). Central processing may further amplify symptom perception through sex-specific interoceptive networks and structural brain differences observed in population-based imaging (32, 33). Together, these observations provide biological plausibility for the higher prevalence in women and explain why neuromodulatory and P2X3-targeted therapies can yield clinically meaningful benefit in female-predominant cohorts (Figure 1).

## Cough hypersensitivity: peripheral and central mechanisms relevant to women

This section synthesizes mechanistic evidence relevant to female predominance in chronic cough, spanning peripheral sensory transduction, central neural processing, and airway–vagal signaling pathways. We examine sex-related differences in cough reflex sensitivity, sensory channel expression, and brainstem–cortical modulation. Together, these mechanisms support the concept that biological sex may influence both the threshold and amplification of cough responses.

## The effect of estrogen on transient receptor potential channels

Estrogen modulates airway sensory excitability through multiple TRP channels, with TRPV1 and TRPA1 being the most extensively studied in the context of cough hypersensitivity (28).

### TRPV1 channel

Estrogen significantly lowers the threshold for capsaicin-induced cough, and women show markedly higher sensitivity to capsaicin than men. During the reproductive years, estrogen enhances phosphorylation at the Ser800 site on the TRPV1 channel via the classical estrogen receptor (ER) signaling pathway, thereby reducing the capsaicin-induced cough threshold by 58% (29, 34). Throughout the menstrual cycle, fluctuations in estrogen levels—particularly the peak in the late follicular phase—augment the TRPV1 channel’s response to capsaicin or high-temperature stimuli, resulting in more intense burning sensations and stronger cough reflexes. Consistent with this, women exhibit 2.7-fold more cough responses to citric acid stimulation during the follicular phase than during the luteal phase (35).

In postmenopausal women, the decline in estrogen levels leads to disinhibition of TRPV1 channels, accompanied by activation of the non-classical GPR30 pathway, which increases the spontaneous firing frequency of airway sensory neurons by 42% (36). Thus, reduced estrogen levels can enhance cough sensitivity by relieving TRPV1 suppression through non-classical estrogen signaling.

The regulation of cough sensitivity by sex hormones is closely related to the prostaglandin/estradiol (PG/E2) ratio. During the luteal phase, an increase in this ratio heightens cough sensitivity, likely because a state of high PG and low E2 counteracts the inhibitory effect of E2 on TRPV1, thereby increasing the excitability of airway afferent nerves (34). Oral contraceptives stabilize TRPV1 function and suppress fluctuations in the excitability of airway afferent nerves through exogenous estrogen, resulting in a consistently elevated baseline cough sensitivity without cyclic variation. These observations suggest that the central nervous system may participate in the regulation of menstruation-related cough sensitivity, in line with the “cough impulse cortical integration” theory (32).

### TRPA1 channel

Estrogen also lowers the activation threshold of the TRPA1 channel, and cold stimuli can intensify coughing via the skin TRPA1 pathway. TRPA1 is highly sensitive to cold and to environmental chemicals such as mustard oil and menthol, which can trigger intense coughing and discomfort. The modulatory effects of estrogen on TRPA1 make women more susceptible to cough symptoms in cold environments, such as during winter or when exposed to cold air (33).

Beyond its effects on ion channels, variation in estrogen levels not only enhances airway nerve sensitivity but may also increase susceptibility to respiratory diseases by modulating the immune system. Estrogen has been shown to regulate T and B cells significantly, and under low-estrogen conditions, immune cell activity is reduced, weakening airway mucosal defense and increasing the risk of pathogen invasion. Furthermore, estrogen plays an important role in modulating airway inflammation: fluctuations in estrogen levels directly promote the release of inflammatory mediators, thereby influencing the degree of airway inflammation and exacerbating cough symptoms (37).

### Progesterone and laryngeal dysfunction

Fluctuations in progesterone levels can lead to significant oedema and venous dilation in the laryngeal mucosa, stimulating rapidly adapting receptors (RARs) and inducing laryngeal hyperresponsiveness (38). Patients typically present with symptoms such as a sensation of a foreign body in the throat, a burning sensation, hoarseness and difficulty swallowing. Even mild stimuli can trigger severe coughing and throat pain (32). Due to unique physiological mechanisms, laryngeal hyperresponsiveness is 2–3 times more prevalent in women than in men (39). This difference is likely to be closely related to hormonal fluctuations, particularly during the menstrual cycle, pregnancy and menopause, when progesterone and other hormone levels fluctuate significantly (40, 41). In postmenopausal women, endogenous progesterone is typically very low; therefore, the clinical relevance of progesterone-mediated laryngeal effects is most applicable to peri-menopause, hormone therapy exposure, or other contexts with measurable progesterone activity.

Fluctuations in progesterone levels can lead to laryngeal mucosal oedema by activating rapidly adapting receptors and exacerbating laryngeal hyperresponsiveness. They can also influence mucosal secretion and increase mucus viscosity, which may worsen throat discomfort and cough through heightened laryngeal irritation. Because eosinophilic airway inflammation and asthma-related traits are frequent aetiologies of chronic cough, hormone-linked Th2/IL-5 signals are most relevant to cough-variant asthma, eosinophilic bronchitis, or overlapping allergic disease rather than to all chronic cough phenotypes. In that context, studies have reported that the frequency of IL-5 and Th2 cells in peripheral blood correlates with airway hyperresponsiveness (AHR; *β* = 0.48, *p* = 0.003), with the strongest association observed during the luteal phase (42). Furthermore, in clinical cohorts the average age of cough onset in women has been reported at ~52.6 years, with onset occurring a few years after the average age of menopause, supporting a potential role for the menopausal transition in cough susceptibility (43).

Evidence remains limited and mostly observational; however, laryngeal discomfort and cough symptoms may fluctuate in women during periods of hormonal transition (e.g., menstrual cycle, pregnancy, and peri−/postmenopause) and in those receiving hormone therapy. Interestingly, women have lower baseline levels of nasal nitric oxide (NO), yet exhibit a significantly stronger response to eotaxin-2 (eosinophil chemotactic factor-2) following allergen stimulation; this response is 1.7 times stronger than in men. This heightened response leads to easier activation of the post-nasal drip-cough pathway (28).

### Central nervous mechanisms

#### Brain imaging evidence

Neuroimaging studies provide important insights into central mechanisms underlying chronic cough, although formal biological sex–stratified analyses remain limited. Most functional MRI (fMRI) and positron emission tomography (PET) studies to date have enrolled mixed-sex cohorts but were not specifically powered to compare women and men (44, 45).

In population-based imaging studies, women comprised a substantial proportion of participants, reflecting the female predominance of chronic cough in the community; however, detailed sex-disaggregated enrollment numbers were not consistently reported across studies. For example, in the Rotterdam population-based cohort examining brain morphometry, women accounted for approximately two-thirds of chronic cough cases, mirroring epidemiologic patterns, but sex-specific subgroup analyses of imaging outcomes were exploratory rather than prespecified (45).

Neuroimaging studies, including fMRI and limited PET investigations, suggest altered central processing in chronic cough. However, formal sex-stratified analyses remain scarce. Functional MRI studies have demonstrated enhanced activation of midbrain regions, including the anterior cingulate cortex and insula, in patients with chronic cough compared with healthy controls. While these studies did not uniformly report sex-stratified activation maps, observational analyses suggest that signal amplification within central cough-processing networks may be more pronounced in women, consistent with their higher cough reflex sensitivity and symptom burden. Importantly, these findings should be interpreted as hypothesis-generating rather than definitive evidence of sex-specific central amplification (46).

Neurophysiological and neuropeptide-related evidence further complements imaging findings. Electromyography (EMG) studies in patients with post-COVID chronic cough suggest vagus nerve dysfunction, characterized by slowed nerve conduction velocity and reduced nerve fiber density (33). In addition, altered functional connectivity between central inhibitory regions, including the anterior cingulate cortex and the nucleus of the solitary tract, has been associated with increased cough frequency, particularly in women.

Overall, although existing neuroimaging data are consistent with a model of enhanced central excitability and impaired inhibitory modulation in women with chronic cough, robust sex-stratified neuroimaging studies remain scarce. Future imaging research should prospectively report sex-specific enrollment, perform predefined sex-based analyses, and integrate hormonal status (e.g., pre- vs. postmenopause) to clarify how biological sex shapes central cough-processing networks (45).

#### Neuropeptides and biological sex differences

The release of substance P (SP) in the nasal mucosa following stimulation has been reported to be higher in women than in men (47). Rather than indicating asthma-specific inflammation, this sex difference may be relevant to cough hypersensitivity through enhanced excitability of upper-airway and laryngeal sensory pathways. Experimental and observational data suggest that estrogen can facilitate mast cell degranulation and neuropeptide release (48), which may secondarily sensitize adjacent sensory nerve endings and lower the cough threshold. When exposed to allergens or physical stimuli, female nasal mucosa may therefore exhibit greater SP release, potentially amplifying sensory signaling and cough responses to otherwise innocuous triggers. Importantly, evidence linking SP predominantly reflects allergic or asthma-related phenotypes, and extrapolation to all chronic cough phenotypes should be made cautiously. Notably, available human observational data suggest that these neuropeptide-related alterations are more frequently observed in female patients; however, most studies were not designed to formally compare biological sex differences.

#### Microbiome dysbiosis

Among patients with persistent cough after COVID-19, small observational studies have reported associations between nasopharyngeal community structure (including higher relative abundance of Staphylococcaceae) and slower symptom resolution (16). However, whether this pattern generalizes to chronic cough from other triggers (e.g., UACS, reflux, eosinophilic inflammation) is unknown; at present these findings should be interpreted as hypothesis-generating rather than a common, established microbiome signature of chronic cough.

### Peripheral nervous mechanisms

#### ATP-P2X3 signaling pathway

The cough reflex is primarily mediated by Aδ fibers and C fibers in the bronchial mucosa. Aδ fibers are sensitive to mechanical and acidic stimuli, while C fibers respond to capsaicin and endogenous mediators. Studies have shown that women generally have a lower cough reflex threshold than men, regardless of whether they are healthy individuals or chronic cough patients, and exhibit stronger cough responses to capsaicin or allyl isothiocyanate-induced stimuli (16). Experimental models suggest increased P2X3-mediated responses in female sensory neurons compared with males and the current amplitude induced by ATP is approximately 41% greater (49). Following airway epithelial damage, ATP is released and rapidly binds to P2X3 receptors on C fibers of the vagus nerve, activating the neural signaling pathway and ultimately triggering a vigorous cough reflex (50). Due to these physiological differences, women are more sensitive to ATP-induced cough responses, resulting in more frequent and intense cough reflexes.

#### Voltage-gated sodium channels (NaV)

The expression of the NaV1.7 channel in female airway sensory neurons is 29% higher than in males. Its inhibitor, XEN402, significantly reduces cough frequency by up to 68% in women, compared to 39% in men (51). The NaV1.7 channel plays a crucial role in the nervous system, particularly in regulating pain perception and the cough reflex. The NaV1.7 and NaV1.8 subtypes are highly expressed in sensory neurons of the airway vagus nerve. When these neurons are activated by irritants such as capsaicin or citric acid, the NaV channels open, facilitating the transmission of neural impulses and subsequently triggering a cough reflex. Inhaling specific NaV inhibitors can effectively block this process, significantly reducing or even completely suppressing cough responses induced by capsaicin and citric acid. This intervention strategy provides new insights into the treatment of chronic cough and related respiratory diseases.

## Hormonal and immune modulators of cough

This section focuses on the role of sex hormones and immune regulation in shaping cough susceptibility and hypersensitivity. We review evidence regarding estrogenic modulation of sensory channels, mast cell activation, neuropeptide release, and inflammatory signaling, while emphasizing areas where direct cough-specific data remain limited. Understanding hormonal–immune interactions may help explain temporal variability across the lifespan and inform hypothesis-driven therapeutic development.

### Mast cells and biological sex hormone interactions

Studies have shown that 82% of mast cells in the nasal mucosa of females are estrogen receptor (ER) positive, and the release of histamine in these cells is 2.1 times higher than in males (52). This receptor is highly expressed on mast cells and estrogen enhances mast cell degranulation and histamine release by activating the receptor and subsequently the TRPV1 channel. Allergen challenge tests have demonstrated a significant linear correlation between elevated tryptase levels in female nasal lavage fluids and TRPV1 activation (*r* = 0.81) (51). This mechanism involves not only chemical signal transmission, but also dynamic changes in the cell membrane and the opening of ion channels. This results in increased intracellular calcium ion concentrations, which promote the release of inflammatory mediators. TRPV1 is expressed on airway vagal C-fiber afferents and plays a well-established role in sensory transduction and cough initiation. TRPV1 expression has also been described in mast cells, where it may influence degranulation responses in experimental settings. These represent distinct cellular compartments within the airway microenvironment, and potential interactions between neurogenic and immunologic pathways remain an area of ongoing investigation (53). For example, mast-cell mediators (including histamine and tryptase) can sensitize airway sensory nerves and lower the cough threshold, but direct evidence that histamine is a dominant trigger in all chronic cough phenotypes is limited; its relevance is likely greatest in allergic rhinitis/UACS, asthma/eosinophilic disease, or mast-cell–rich airway inflammation.

Animal experiments have confirmed that female rats exhibit a more intense response to capsaicin in mast cells. Observations of skin tissue revealed that mast cells in female rats degranulated and released large amounts of histamine rapidly after exposure to capsaicin, exhibiting a significantly enhanced inflammatory response (29). These findings suggest that females may have unique physiological mechanisms that lead to stronger immune and inflammatory responses when exposed to the same stimuli.

### Obstructive sleep apnea

Obstructive sleep apnoea (OSA) is a common sleep disorder involving repeated partial or complete blockage of the upper airway during sleep. Research indicates that postmenopausal women are at an increased risk of developing OSA due to a significant decline in estrogen levels (36). The reduction in estrogen affects metabolic and immune functions, and may also lead to a decrease in upper airway muscle tone, thereby exacerbating airway obstruction.

OSA induces chronic cough through multiple mechanisms. Firstly, OSA can cause gastro-esophageal reflux, whereby gastric acid flows back into the esophagus and pharynx, triggering a cough. In both pre- and postmenopausal women, a decline in estrogen levels results in a 20–30% reduction in lower esophageal sphincter tone, as well as delayed gastric emptying. This significantly increases the frequency of reflux events. Twenty-four-hour multichannel intraluminal impedance-pH (MII-pH) monitoring has shown that a DeMeester score >14.7 in women is associated with decreased cough sensitivity (*r* = 0.62, *p* < 0.01) (54, 55).

Secondly, the release of inflammatory mediators triggered by OSA is another mechanism. These chemical substances can induce local and systemic inflammatory responses, continuously stimulating the airways and leading to a chronic cough (56, 57). Furthermore, nocturnal hypoxaemia in OSA patients exacerbates airway inflammation, thereby promoting the cough reflex. Some observational data suggest that estrogen supplementation may alleviate OSA-related symptoms; however, its impact on chronic cough remains insufficiently studied (58). This suggests that estrogen plays a critical role in maintaining airway homeostasis and provides new insights for clinical treatment. Regulation of estrogen levels influences not only the pathogenesis of OSA, but also modulates mast cell activity, thereby directly intervening in the sensitivity of the cough reflex. Further research into the interactions between estrogen, mast cells and the TRPV1 channel is expected to lead to the development of novel therapeutic strategies for female OSA patients, improving treatment efficacy and enhancing quality of life for these patients (Figure 1).

Although obstructive sleep apnoea (OSA) is more prevalent in men overall, emerging evidence suggests that hormonal status may modulate cough susceptibility in women with OSA. Estrogen influences upper airway neuromuscular tone, inflammatory signaling, and sensory neural excitability; declining estrogen levels, particularly after menopause, may increase airway vulnerability and cough reflex sensitivity (59). Consequently, the epidemiology of OSA and the expression of cough symptoms may diverge, reflecting differences in hormonal modulation rather than simple disease prevalence. However, direct mechanistic studies examining sex-specific cough responses in OSA remain scarce and warrant further investigation.

Other contributors that may influence the observed predominance include smaller airway caliber relative to body size, age-related hormonal transitions (e.g., peri- and post-menopause), comorbid conditions that lower cough threshold (e.g., chronic rhinosinusitis), and sex-specific central nervous system processing that shapes cough perception and control. Recognizing these factors—alongside potential differences in health-seeking behavior and prescribing—adds balance and helps interpret heterogeneity across studies (43, 56).

## Comorbidities and triggers with differential impact

This section examines comorbid conditions and environmental triggers that may disproportionately affect women with chronic cough, including reflux-related syndromes, upper airway dysfunction, asthma phenotypes, and post-infectious states. We discuss how biological susceptibility intersects with behavioral, occupational, and psychosocial factors to influence symptom burden. Recognizing these interactions is critical for differentiating cause-directed management from treatment of cough hypersensitivity itself.

### Neuromodulators

Gabapentin and pregabalin: Randomized controlled trials have demonstrated that gabapentin and pregabalin can improve cough-specific quality of life and reduce cough severity in patients with refractory chronic cough (60, 61). However, most studies were not powered to detect sex-stratified differences in treatment response; therefore, evidence for female-specific superiority remains insufficient.

Amitriptyline: This tricyclic antidepressant is particularly effective in treating coughs caused by viral infections via vagal nerve mediation. It works by modulating neural transmission pathways, significantly reducing the frequency and intensity of the cough reflex (56, 62, 63).

### Interventional neuromodulation: superior laryngeal nerve block

Superior laryngeal nerve (SLN) block has emerged as a targeted interventional option for selected patients with refractory chronic cough, particularly those with prominent laryngeal hypersensitivity or neurogenic cough features. The procedure typically involves unilateral or bilateral injection of a local anesthetic, with or without corticosteroid, at the level of the internal branch of the SLN, aiming to attenuate aberrant afferent sensory signaling from the larynx.

Small prospective studies and case series have reported clinically meaningful reductions in cough frequency and severity, as well as improvements in cough-related quality of life, with acceptable short-term safety profiles (39). SLN block is generally considered when behavioral cough suppression therapy and pharmacological neuromodulation fail or are poorly tolerated, and it is increasingly incorporated into multidisciplinary cough management pathways in specialized centers.

Although women constitute a substantial proportion of reported cohorts—likely reflecting the female predominance of laryngeal hypersensitivity phenotypes—existing studies were not designed to assess sex-specific treatment effects, and biological sex has not been evaluated as a predefined predictor of response. Accordingly, SLN block should currently be regarded as a phenotype-driven rather than sex-specific intervention, highlighting the need for future sex-stratified interventional studies.

## Therapeutic landscape and safety considerations

This section reviews current and emerging therapies for chronic cough, clearly distinguishing etiology-directed treatments from antitussive strategies targeting cough hypersensitivity. We evaluate evidence for behavioral therapy, neuromodulators, P2X3 antagonists, and other mechanism-oriented interventions, while considering potential sex-specific efficacy and safety profiles. These therapeutic insights are interpreted in light of the mechanistic pathways discussed earlier in the review.

P2X receptors are ATP-gated trimeric ion channels that can assemble as homotrimeric P2X3 receptors or heterotrimeric P2X2/3 receptors. Homomeric P2X3 receptors are highly expressed on vagal sensory fibers innervating the airways and are thought to play a key role in ATP-driven cough hypersensitivity. By contrast, heteromeric P2X2/3 receptors are abundantly expressed on gustatory afferents and are essential for normal taste perception. Antagonists that non-selectively block both P2X3 homotrimers and P2X2/3 heterotrimers therefore tend to produce a trade-off between antitussive efficacy and taste-related adverse events, whereas newer, more P2X3-selective compounds are designed to inhibit P2X3 homotrimers while largely sparing P2X2/3 heterotrimers, with the aim of maintaining efficacy and reducing dysgeusia. In addition to gefapixant, eliapixant, and camlipixant, other selective P2X3 antagonists such as sivopixant have been evaluated in phase 2 trials, although efficacy signals have been inconsistent and further development remains uncertain (10, 64–71).

## Methodological gaps and biological sex-stratified research priorities

This section identifies methodological and conceptual gaps that limit definitive conclusions regarding sex differences in chronic cough. We highlight the scarcity of sex-stratified analyses, limited mechanistic trials powered for biological sex comparisons, and the need for standardized phenotyping. Addressing these gaps will require integrative, multidisciplinary research frameworks and more consistent reporting of sex-disaggregated outcomes.

### Speech therapy

Behavioral cough suppression therapy, often delivered by speech and language therapists, has emerged as an effective non-pharmacological intervention for refractory and unexplained chronic cough. Importantly, its efficacy has been demonstrated specifically in chronic cough populations rather than being extrapolated solely from studies of other laryngeal dysfunctions such as paradoxical vocal fold motion.

A randomized controlled trial has shown that speech therapy–based interventions significantly improve clinical cough outcomes. In a landmark randomized controlled study, Vertigan et al. demonstrated that a structured speech pathology program led to significant reductions in cough frequency and severity, alongside improvements in cough-related quality of life, compared with control interventions (72). The recent meta-analysis by Yi et al. (73) provides a comprehensive overview of specific speech therapy components used across studies; however, the therapeutic benefit of these interventions is primarily grounded in evidence from randomized controlled trials directly assessing cough-related endpoints. Collectively, these data support speech therapy as a core component of multidisciplinary management for chronic cough, independent of coexisting laryngeal disorders.

However, most randomized trials and observational studies have not reported sex-stratified efficacy analyses. It therefore remains uncertain whether treatment response differs by biological sex, or whether differences in laryngeal sensitivity, neuromodulation, or symptom perception might influence therapeutic outcomes. Future studies incorporating predefined sex-disaggregated analyses may help clarify whether speech pathology–based interventions have sex-specific effects.

### Anti-inflammatory therapy

Anti-inflammatory therapies, such as intranasal corticosteroids or inhaled corticosteroids, improve cough primarily when directed at specific treatable traits (e.g., allergic rhinitis, chronic rhinosinusitis, cough-variant asthma, or eosinophilic bronchitis). However, they are not expected to benefit chronic cough from unrelated causes (e.g., refractory cough hypersensitivity without UACS, isolated reflux-related cough, or medication-induced cough), and their use should be framed as etiology-specific rather than universal therapy (18, 35).

Although women more frequently present with certain inflammatory or upper-airway–dominant phenotypes, clinical trials have not demonstrated consistent sex-based differences in treatment efficacy, nor has sex been incorporated as a predictor variable in treatment algorithms. Therefore, the apparent female predominance among responders likely reflects differences in underlying comorbidity patterns, rather than intrinsic sex-dependent pharmacologic responsiveness.

### Psychological interventions

Psychological interventions, including cognitive behavioral therapy, address anxiety, depression, and heightened symptom perception frequently associated with chronic cough. While psychosocial comorbidities are reported more commonly in women with chronic cough, existing interventional studies do not provide evidence that biological sex modifies the therapeutic effect of psychological interventions.

Accordingly, psychological support should be regarded as an adjunctive, symptom-oriented strategy applicable to both sexes, with sex-related differences influencing comorbidity prevalence and illness perception, rather than treatment efficacy per se.

Psychological interventions, including cognitive behavioral therapy (CBT), can alleviate anxiety and depression associated with chronic cough and improve health-related quality of life by enhancing emotional regulation, coping strategies, and treatment confidence (74). Approaches incorporating psychological resilience principles further help patients manage stress, adjust illness perceptions, and redirect attention away from cough-related distress (75). Supportive communication, reliable health information, and timely referral for professional psychological care remain important when symptoms persist or impair daily functioning (44, 76).

Importantly, across non-pharmacological and anti-inflammatory interventions for chronic cough, biological sex has rarely been incorporated as a predefined effect modifier, underscoring a major methodological gap in the current evidence base.

### Precision targeted therapy

#### TRP channel inhibitors

TRP channel inhibitors: High-selectivity TRPV1 and TRPA1 antagonists remain an area of active investigation for cough hypersensitivity, but clinical translation has been challenging and efficacy signals have been inconsistent across programs. Accordingly, we now frame multi-target combination strategies (including analgesic-pathway intermediates such as AM404) as preclinical hypotheses rather than established clinical approaches, and we emphasize the need for robust, cough-specific randomized trials before recommending these strategies in practice.

#### Estrogen receptor modulators

Estrogen receptor modulators (SERMs): While estrogen signaling can influence mast-cell biology and neuro-immune interactions in experimental systems, there is currently no high-quality clinical-trial evidence supporting SERMs as an antitussive therapy across chronic cough phenotypes. At present, any consideration of SERMs should be restricted to research settings or to standard indications unrelated to cough, with careful attention to risks and benefits.

#### Public health strategies

Public health strategies for chronic cough should recognize its substantial psychosocial and physical burden, particularly among women. Chronic cough is associated with significant impairment in daily functioning, social participation, and work productivity, and these impacts are often more pronounced in female patients. One well-documented consequence is cough-related stress urinary incontinence, which disproportionately affects women and contributes to embarrassment, activity avoidance, and delayed healthcare seeking.

In addition to physical morbidity, women with chronic cough frequently experience psychological distress, including anxiety and depressive symptoms, which are closely linked to cough severity and chronicity. Population-based and clinic-based studies have shown high rates of anxiety and depression in chronic cough cohorts, with women reporting greater symptom burden and poorer health-related quality of life. These psychosocial effects may further amplify cough perception and perpetuate symptom persistence through heightened central sensitivity.

From a public health perspective, effective strategies should therefore include early identification of cough-related comorbidities, routine screening for urinary incontinence and mental health disorders, patient education to reduce stigma, and timely referral to multidisciplinary care. Addressing these broader consequences is essential to reducing disease burden and improving outcomes for women with chronic cough.

However, in a large pan-European community survey, only 30% of more than 1,100 patients with chronic cough reported that their physicians had “thoroughly addressed their cough issue” (77). Multi-channel advocacy is therefore needed to raise public awareness of the burden of chronic cough, promote evidence-based prevention and treatment, and build a comprehensive health management network (Figure 1).

## Future direction

This section outlines emerging strategies to advance precision medicine in chronic cough. Biomarker-guided phenotyping, artificial intelligence–assisted modeling, and novel digital and genetic approaches may help refine diagnosis and personalize treatment. However, these technologies remain largely exploratory and require rigorous validation. Future progress will depend on integrating multidisciplinary research within sex-stratified and intersectional frameworks to ensure clinically meaningful and equitable improvements in care.

### Mechanism-based personalized treatment

Identifying biomarkers is crucial for diagnosing and treating chronic cough. Using indicators such as fractional exhaled nitric oxide (FENO) and induced sputum eosinophil counts makes it possible to identify cough subtypes, such as hormone-sensitive or neurogenic cough. This provides more accurate diagnostic guidance and helps to formulate personalized treatment plans (78). FENO testing can precisely assess airway inflammation, while induced sputum eosinophil counts can effectively differentiate between different cough subtypes (63, 79). Combining these two tests enables clinicians to select the most appropriate treatment, thereby improving therapeutic outcomes.

Future research should expand sample sizes to further validate the clinical value of FENO and sputum eosinophil counts, thus advancing the development of precision medicine for chronic cough (80). Additionally, integrating genetic testing could help to identify patient sensitivity to specific treatment plans, optimizing therapeutic strategies and improving treatment success rates (81). Investigating the interactions between genetics and environmental factors will contribute to our understanding of the pathogenesis of chronic cough and provide stronger support for precision medicine. At the same time, establishing multidisciplinary collaboration platforms and integrating resources will promote the development of a comprehensive management system for chronic cough and further enhance patients’ quality of life.

### Artificial intelligence-assisted diagnosis

By combining symptom scores (e.g., HARQ) with multi-omics data, advanced AI technology can be used to construct predictive models and rapidly and accurately analyze large volumes of data, offering optimal treatment recommendations for patients (1). AI application not only enhances diagnostic accuracy and efficiency, but also continuously improves algorithms through learning, promoting the rational allocation of medical resources.

With the integration of multimodal data, AI will be able to assess a patient’s health status more comprehensively, assisting in the formulation of personalized treatment plans and significantly improving patient outcomes and advancing healthcare. AI-assisted chronic cough management will enable intelligent management at every stage, from prevention to treatment, with potential to improve patient adherence. Through real-time monitoring of disease progression, AI can dynamically adjust treatment plans to ensure precise and effective treatment. Furthermore, AI can enhance doctor-patient communication, offer personalized health guidance and establish a comprehensive chronic cough management system, thereby providing patients with a superior healthcare experience.

### Gene editing and digital therapies

#### CRISPR-Cas9

Gene editing and digital therapeutics: Wearable sensors (e.g., combining cardiorespiratory signals and upper-airway impedance or acoustics) are being explored to detect cough events and potentially anticipate periods of heightened cough risk, but external validation, generalizability across causes of chronic cough, and clinical utility remain uncertain. These approaches should be considered emerging research tools rather than ready-to-implement clinical solutions.

Future research should adopt explicitly sex-stratified and intersectional designs to examine how biological sex interacts with race and other demographic characteristics in shaping chronic cough risk, clinical presentation, treatment response, and underlying pathophysiology. Achieving meaningful progress will require the coordinated integration of epidemiology, translational neuroscience, and rigorously designed interventional trials to determine whether sex-informed and equity-oriented therapeutic strategies can improve outcomes in chronic cough.

## Conclusion

Women experience a higher incidence and burden of chronic cough, with middle-aged and older (peri−/postmenopausal) women over-represented in refractory and unexplained chronic cough cohorts. Available evidence supports a cough hypersensitivity framework in which peripheral sensory pathways (e.g., TRP channels, ATP–P2X3 signaling, and NaV channels), laryngeal dysfunction, and central cough-control networks contribute to symptom persistence, while comorbid treatable traits (UACS, eosinophilic airway disease, reflux) remain essential to identify and manage. Hormone-related mechanisms should be interpreted in a life-course context: cyclical fluctuations may modulate symptoms in premenopausal states, whereas the menopausal transition may interact with neural excitability, mucosal biology, and comorbidity clustering. Therapeutically, management of chronic cough in women should remain etiology-directed, with careful identification of treatable traits such as upper airway cough syndrome, eosinophilic airway disease, and reflux-related cough. For refractory or unexplained chronic cough, behavioral cough suppression therapy and neuromodulatory approaches are supported by current evidence. Although emerging antitussive agents targeting sensory pathways (e.g., purinergic P2X3 signaling) show clinical promise, existing trials were not designed to evaluate sex-specific efficacy or safety. As such, their relevance to female-predominant cough populations should be interpreted cautiously, highlighting the need for future trials incorporating predefined biological sex–stratified analyses. Key research priorities include standardized cough outcomes, robust sex- and menopause-stratified analyses, mechanistic studies that distinguish phenotype-specific pathways, and pragmatic trials that evaluate real-world effectiveness and safety in women.
